# Mechanistic target of rapamycin and an extracellular signaling-regulated kinases 1 and 2 signaling participate in the process of acetate regulating lipid metabolism and hormone-sensitive lipase expression

**DOI:** 10.5713/ab.21.0341

**Published:** 2021-10-29

**Authors:** Yujuan Li, Chunyan Fu, Lei Liu, Yongxu Liu, Fuchang Li

**Affiliations:** 1 Department of Animal Science, Shandong Agricultural University, Taian, Shandong 271018, China; 2 Qingdao Kangda Food Co., LTD., Qingdao, Shandong 266555, China

**Keywords:** Acetate, Extracellular Signaling-regulated Kinases 1 and 2 (ERK1/2), Lipid Deposition, Mechanistic Target of Rapamycin (mTOR), Rabbit Adipose-derived Stem Cells

## Abstract

**Objective:**

Acetate plays an important role in host lipid metabolism. However, the network of acetate-regulated lipid metabolism remains unclear. Previous studies show that mitogen-activated protein kinases (MAPKs) and mechanistic target of rapamycin (mTOR) play a crucial role in lipid metabolism. We hypothesize that acetate could affect MAPKs and/or mTOR signaling and then regulate lipid metabolism. The present study investigated whether any cross talk occurs among MAPKs, mTOR and acetate in regulating lipid metabolism.

**Methods:**

The ceramide C6 (an extracellular signaling-regulated kinases 1 and 2 [ERK1/2] activator) and MHY1485 (a mTOR activator) were used to treat rabbit adipose-derived stem cells (ADSCs) with or without acetate, respectively.

**Results:**

It indicated that acetate (9 mM) treatment for 48 h decreased the lipid deposition in rabbit ADSCs. Acetate treatment decreased significantly phosphorylated protein levels of ERK1/2 and mTOR but significantly increased mRNA level of hormone-sensitive lipase (HSL). Acetate treatment did not significantly alter the phosphorylated protein level of p38 MAPK and c-Jun aminoterminal kinase (JNK). Activation of ERK1/2 and mTOR by respective addition in media with ceramide C6 and MHY1485 significantly attenuated decreased lipid deposition and increased HSL expression caused by acetate.

**Conclusion:**

Our results suggest that ERK1/2 and mTOR signaling pathways are associated with acetate regulated *HSL* gene expression and lipid deposition.

## INTRODUCTION

Volatile fatty acids (VFA, acetate, propionate, and butyrate) are produced by the anaerobic fermentation of unabsorbed carbohydrates in the rabbit cecum [1]. The VFA are transported across the apical and the basolateral membranes of colonocytes. Acetate as major VFA in rabbit gut (70% to 80%), is an important nutrient and acts as a signaling molecule in various cellular processes [2,3]. Acetate could affect insulin secretion, gastrointestinal motility, and blood flow, and cell proliferation, apoptosis, and differentiation [4,5]. Previous studies showed that acetate was a substrate for lipogenesis and stimulated adipocyte differentiation [6]. In rabbits, acetate treatment decreased scapular fat yield and triglyceride concentration in liver, fat adipose tissue and plasma [7]. In porcine stromal vascular fraction, acetate treatment enhanced adipocyte differentiation [8]. Acetate could regulate the gene expression of limiting enzymes of fatty acid oxidation (carnitine palmitoyl transferase 1, CPT1) and synthesis (fatty acid synthase, FAS) [9]. But the mechanism of acetate regulating lipid metabolism is still unclear.

Mechanistic target of rapamycin (mTOR) signaling pathway responds to nutrient and growth factor [10]. mTOR signaling is associated with the lipogenesis. The inhibition of mTOR using rapamycin abrogates adipocyte differentiation in various cell lines *in vitro* [11–13]. CCAAT/enhancer-binding proteins (C/EBPs) and peroxisome proliferator-activated receptor-γ (PPARγ) were the important transcription factors in regulating the differentiation of preadipocytes into adipocytes [14,15]. The previous studies have shown that rapamycin could inhibit the expression of C/EBPs and PPARγ in 3T3-L1 and 3T3-F442A cells [11]. Mitogen-activated protein kinases (MAPKs), including extracellular regulated kinase 1/2 (ERK1/2), p38 and c-Jun-N-terminal kinase (JNK), play a crucial role in adipocyte differentiation [16]. The inhibition of ERK1/2 or p38 reduced adipocyte differentiation [17,18]. In 3T3-L1 cells, ERK activity is necessary for the expression of C/EBPα and PPARγ [19]. However, the role of mTOR and MAPKs signaling in acetate-regulated adipocyte metabolism remains unclear.

We hypothesize acetate could regulate MAPKs and/or mTOR signaling and then affect lipid metabolism. In our study, we investigated the effect of acetate on mTOR, and MAPKs signaling expression in rabbit adipose-derived stem cells (ADSCs) and examined the major signaling pathway of acetate-mediated lipid metabolism.

## MATERIALS AND METHODS

### Animal welfare statement

The authors declare that they have complied with the ethics policy described int the journal’s author guide. All study procedures were approved by the Shandong Agricultural University Animal Care and Use Committee (SDAUA-2019-088) and were in accordance with the Guidelines for Experimental Animals established by the Ministry of Science and Technology (Beijing, China).

### Rabbit adipose-derived stem cells culture and treatments

ADSCs (Cyagen Biosciences, Guangzhou, China) were seeded in 6-well plates and cultured in Dulbecco’s modified eagle’s medium (DMEM)/F12 (Gibco, Inchinnan, Scotland), supplemented with 10% fetal bovine serum (Biological Industries, Beit-Haemek, Israel) and 100 IU/mL penicillin and streptomycin (growth medium) (Gibco, Scotland) at 37°C. The medium was replenished every 48 h. After 3-day incubation, the cells were incubated with differentiation medium (growth medium supplemented with insulin, dexamethasone, rosiglitazone and 3-isobutyl-1-methylxanthine) to induce adipogenic differentiation for another 3 d.

#### Experiment 1

After induction in differentiation medium for 3 d, the cells were then maintained in DMEM/F12 medium, supplemented with saline (control) or 9 mM acetate (Sigma-Aldrich, St. Louis, MO, USA). Acetate concentration was determined according to previous studies [8,20,21]. The cells were collected after treatment for 48 h.

#### Experiment 2

To clarify the role of ERK signaling in acetate-regulated lipid metabolism induced, the 3-day differentiated cells were treated with 10 μM Ceramide C6 (an ERK1/2 activator, Santa Cruz Technology, Santa Cruz, CA, USA) or vehicle (dimethyl sulfoxide) for 12 h [22]; the cells were then given a treatment of either acetate (9 μM) or saline for 48 h before collected.

#### Experiment 3

To determine the role of mTOR signaling in acetate-regulated lipid metabolism, the cells were maintained in DMEM/F12 with 10 μM MHY1485 (a mTOR activator, Merck Millipore, Billerica, MA, USA) or vehicle (dimethyl sulfoxide) for 10 h [23]. Afterwards, the cells were given a treatment of either acetate (9 μM) or saline for 48 h and collected later.

### Oil red O staining

The accumulation of cytoplasmic lipid droplets was visualised by oil red O staining according to the protocol [24]. Briefly, cells were rinsed twice with phosphate buffered saline (PBS) and subsequently fixed with 10% formalin in PBS for 1 h at room temperature. Then, the cells were washed twice with PBS and stained with oil red O working solution prepared in 60% isopropyl (Sigma-Aldrich, USA) from a stock of 3.5 mg/mL for 1 h. The cells were then washed 3 to 5 times with PBS. Hematoxylin solution was used to stain the nucleus of a cell for 10 s, and then the cells were photographed using a Nikon optical microscope equipped with a Nikon camera (Nikon Instruments Inc., Melville, NY, USA). The volume density of each oil red O positive fiber within the cell was determined by Weibel’s point-counting method [25].

### RNA extraction and quantitative real-time polymerase chain reaction analyses

Gene expression was quantified using quantitative real-time polymerase chain reaction (PCR) with SYBR Green I labeling. The total RNA extraction, reverse transcription and PCR were performed as the previous description [26]. Total RNA was isolated using the guanidinium isothiocyanate method with Trizol Reagent (Invitrogen, San Diego, CA, USA). The quality of the RNA was tested by electrophoresis on an agarose-gel and the quantity of the RNA was determined with biophotometer (Eppendorf, Hamburg, Germany). RT reactions (10 μL) consisted of 500 ng total RNA, 5 mmol/L MgCl_2_, 1 μL RT buffer, 1 mmol/L dNTP, 2.5 U AMV, 0.7 nmol/L oligo d(T) and 10 U Ribonuclease inhibitor (TaKaRa Biotechnology, Co., Ltd. Dalian, China). Real-time PCR analysis was conducted using the Applied Biosystems 7500 Real-time PCR System (Applied Biosystems, Foster, CA, USA). Each RT-reaction served as a template in a 20 μL PCR reaction containing 0.2 μmol/L of each primer and SYBR green master mix (Takara Biotechnology, Co., Ltd. China). Primer-set sequences are described in Table 1. Real-time PCR reactions were performed at 95°C for 10 s, followed by 40 cycles at 95°C for 5 s and 60°C for 34 s. SYBR green fluorescence was detected at the end of each cycle to monitor the amount of PCR product. The mRNA levels of the target genes were normalized to glyceraldehyde 3-phosphate dehydrogenase and β-actin (ΔCT) [27]. The ΔCT was calibrated against an average from the control checks. The number of target molecules relative to the control was calculated using 2^−ΔΔCT^. Therefore, all the gene transcription results are reported as the n-fold difference relative to the calibrator. Specificity of the amplification product was verified.

### Protein preparation and western blot analyses

The rabbit ADSCs were homogenized in 0.2 mL of lysis buffer (Beyotime, Jiangsu, China) and kept on ice during the trial procedure. The homogenate was centrifuged at 12,000 g for 5 min at 4°C, and the supernatant was collected. Protein concentration was assayed using a bicinchoninic acid assay kit (Beyotime, China) according to the manufacturer’s protocol. Aliquots of 25 μg of protein were separated with 7.5% to 10% sodium dodecyl sulfate polyacrylamide gels (Bio-Rad Inc, Richmond, CA, USA) according to the previous method [28,29], and the proteins were then transferred onto a polyvinylidene fluoride membrane (Millipore, USA) at 200 mA for 2 h in a Tris-glycine buffer with 20% anhydrous ethanol at 4°C. The membranes were blocked with western blocking buffer (Beyotime, China) for 1 h at room temperature. The membranes were then probed with primary antibodies at 4°C with gentle shaking overnight. The primary antibodies used were anti-p-p38 MAPK^Thr180/Tyr182^, anti-p38 MAPK, anti-p-ERK1/2^Thr202/Tyr204^, anti-ERK1/2, anti-p-JNK^Thr183/Tyr185^, anti-JNK, anti-mTOR, anti-p-mTOR^Ser2448^ (Cell Signaling Technology, Trask Lane Danvers, MA, USA) and anti-Tubulin (Beyotime, China). After being washed, the membranes were incubated with horseradish peroxidase-linked anti-rabbit or anti-mouse secondary antibodies for 4 h at 4°C. The membranes were then visualized by exposure to Hyperfilm Electro-Chemi-Luminescence (Beyotime, China). Western blots were developed and quantified using Bio Spectrum 810 with Vision Works LS 7.1 software (UVP LLC, Upland, CA, USA).

### Statistical analysis

All data were expressed as the mean±standard error of the mean. The data were analyzed by t-test with SAS software in experiment 1 (n = 8 independent replicates). The data were analyzed by one-way analysis of variance in experiment 2 and 3 (n = 8 independent replicates). Multiple comparisons between the groups were performed by the Tukey method. Less than 0.05 p value was considered statistically significant.

## RESULTS

### Effect of acetate on lipid metabolism and related signaling expression in rabbit ADSCs (Experiment 1)

**Figure 1:**
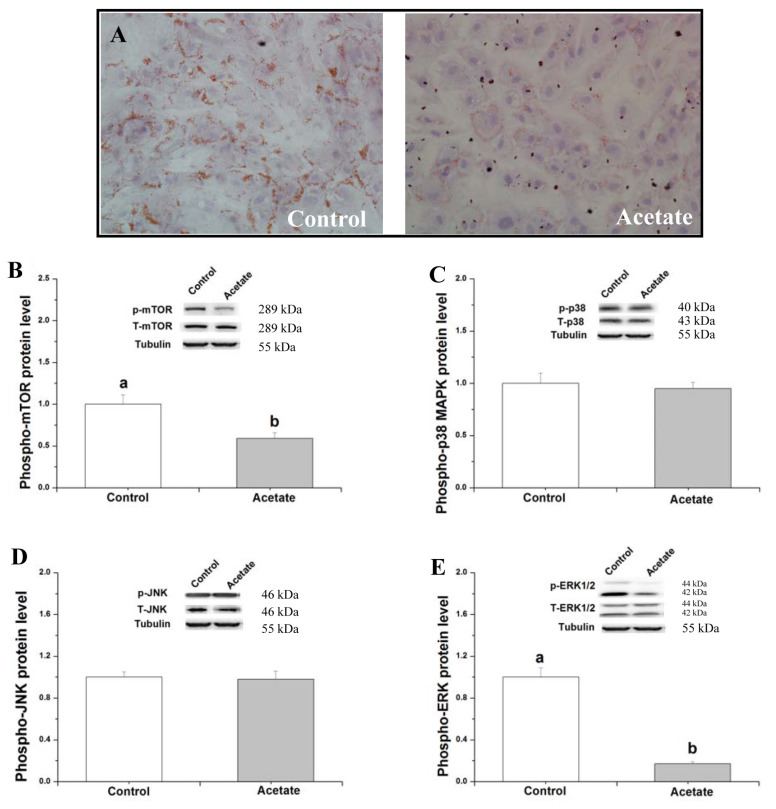
Effects of acetate on lipid metabolism and related signaling expression in rabbit adipose-derived stem cells (ADSCs). After induction in differentiation medium for 3 d, the cells were then maintained in Dulbecco’s modified eagle’s medium/F12 medium, supplemented with saline (control) or 9 mM acetate for 48 h. (A) Representative images of oil red O-stained cells (200× magnification); (B–E) protein levels of mTOR, p38 MAPK, JNK, and ERK1/2. Data are presented as means±standard error of the mean (n = 8 independent replicates). mTOR, mechanistic target of rapamycin; p38 MAPK, p38 mitogen-activated protein kinase; JNK, c-Jun-N-terminal kinase; ERK1/2, extracellular signaling-regulated kinases 1 and 2. ^a,b^ Bars with different letters are significantly different (p<0.05).

To test whether acetate plays a role in adipogenesis in rabbit ADSCs, the lipid droplet concentration was measured by oil red O staining (Figure 1A). It indicated that 9 mM acetate treatment for 48 h decreased the lipid droplet concentration compared with the control. To explore the related signaling pathways of acetate-mediated lipid metabolism, the phosphorylated protein levels of mTOR, p38 MAPK, JNK, and ERK were measured after acetate treatment in rabbit ADSCs. Compared with the control, acetate treatment significantly decreased the phosphorylated protein levels of mTOR and ERK (Figure 1B, 1E; p<0.05), but did not significantly alter the phosphorylated protein levels of p38 MAPK and JNK (Figure 1C, 1D; p>0.05).

### Effects of ERK1/2 signaling activation on lipid droplet deposition and gene expression related to fat metabolism after acetate treatment (Experiment 2)

**Figure 2:**
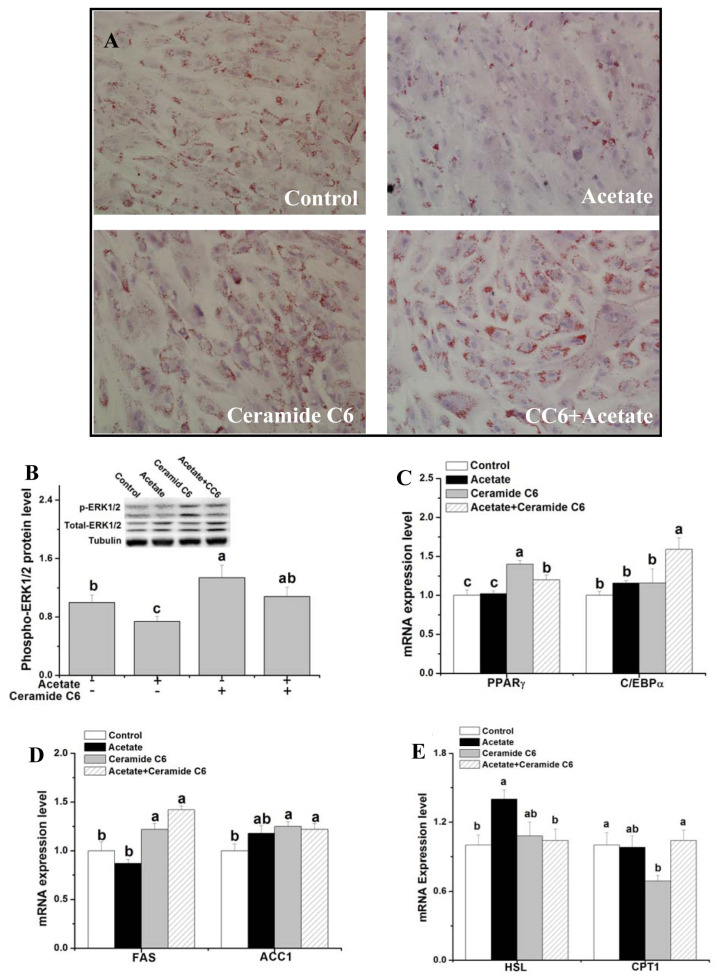
Effects of ERK1/2 signaling activation on lipid droplet deposition and gene expression related to fat metabolism after acetate treatment. After induction in differentiation medium for 3 d, rabbit ADSCs were cultured in the presence of 10 μM Ceramide C6 or vehicle (dimethyl sulfoxide) for 12 h and then treated with acetate (9 μM) or saline for 48 h. (A) Representative images of oil red O-stained cells (200× magnification); (B) p-ERK1/2 protein expression; (C–E) Relative mRNA levels of PPARγ, C/EBPα, FAS, ACC1, HSL, and CPT1. Data are presented as means±standard error of the mean (n = 8 independent replicates). ERK1/2, extracellular signaling-regulated kinases 1 and 2; PPARγ, peroxisome proliferator-activated receptor-γ; C/EBPα, CCAAT/enhancer-binding protein α; FAS, fatty acid synthase; ACC1, acetyl-CoA carboxylase 1; HSL, hormone-sensitive lipase; CPT1, carnitine palmitoyl transferase 1. ^a–c^ Bars with different letters are significantly different (p<0.05).

To verify the function of ERK1/2 signaling in acetate-mediated lipid metabolism, the lipid droplet concentration and gene expression related to lipid metabolism were measured after ERK1/2 activator (ceramide C6) and acetate treatments in rabbit ADSCs. Compared with the control, ceramide C6 treatment significantly increased the lipid droplet concentration, phosphor-ERK protein level and PPARγ, FAS, and acetyl-CoA carboxylase 1 (ACC1) mRNA levels (Figure 2A–2D; p<0.05), but significantly decreased the CPT1 mRNA level (Figure 2E; p<0.05). Compared with the control, only ceramide C6 treatment had no significant effect on *C/EBPα* and hormone-sensitive lipase (*HSL*) genes expression (Figure 2C, 2E; p>0.05). Pretreatment with ceramide C6 significantly attenuated acetate-inhibited lipid droplet deposition, phosphor-ERK1/2 protein expression (Figure 2A, 2B; p<0.05) and acetate-induced *HSL* genes expression (Figure 2E; p<0.05). Compared with the control, concurrent treatment of acetate and ceramide C6 increased *PPARγ*, *C/EBPα*, *FAS*, and *ACC1* genes expression (Figure 2C, 2D; p<0.05).

### Effects of mTOR signaling activation on lipid droplet deposition and gene expression related to fat metabolism after acetate treatment (Experiment 3)

**Figure 3:**
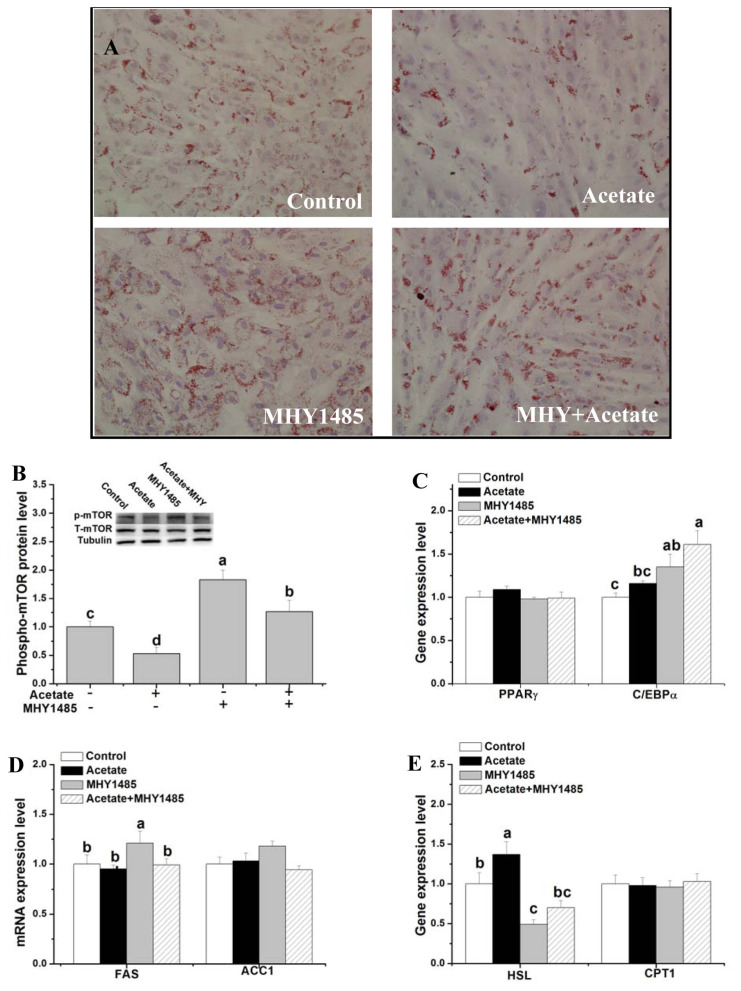
Effects of mTOR signaling activation on lipid droplet deposition and gene expression related to fat metabolism after acetate treatment. After induction in differentiation medium for 3 d, rabbit ADSCs were cultured in the presence of 10 μM MHY1485 or vehicle (dimethyl sulfoxide) for 10 h and then treated with acetate (9 μM) or saline for 48 h. (A) Representative images of oil red O-stained cells (200× magnification). (B) p-mTOR protein expression. (C–E) Relative mRNA level of PPARγ, C/EBPα, FAS, ACC1, HSL, and CPT1. Data are presented as means±standard error of the mean (n = 8 independent replicates). mTOR, mechanistic target of rapamycin; PPARγ, peroxisome proliferator-activated receptor-γ; C/EBPα, CCAAT/enhancer-binding proteins α; FAS, fatty acid synthase; ACC1, acetyl-CoA carboxylase 1; HSL, hormone-sensitive lipase; CPT1, carnitine palmitoyl transferase 1. ^a–d^ Bars with different letters are significantly different (p<0.05).

To determine the role of mTOR signaling in acetate-regulated lipid metabolism, the lipid droplet concentration and gene expression levels related to lipid metabolism were measured after mTOR activator (MHY1485) and acetate treatments in rabbit ADSCs. Compared with control, the cells with MHY1485 treatment had an increase in lipid droplet concentration, phosphor-mTOR protein level and C/EBPα and FAS mRNA levels (Figure 3A–3D; p<0.05), and a decrease in HSL mRNA level (Figure 3E; p<0.05). The inhibition of acetate on lipid droplet deposition and phospho-mTOR protein expression as well as the stimulation of *HSL* gene expression was significantly attenuated by pretreatment with MHY1485 (Figure 3A, 3B, 3E; p<0.05). Only activation of mTOR had no significant effect on PPARγ, ACC1, and CPT1 mRNA levels compared with the control (Figure 3C–3E; p>0.05). Compared with the control, concurrent treatment of acetate and ceramide C6 increased the phosphor-mTOR protein level and *C/EBPα* gene expression (Figure 3B, 3C; p<0.05) (Supplementary Figure S1).

## DISCUSSION

### Effect of acetate on lipid metabolism in rabbit ADSCs

The VFA can be used for de novo synthesis of lipids and glucose and acts as signaling molecules in energy expenditure and homoeostasis [30], while the effect of acetate on lipid metabolism is consistent. The previous studies shown that acetate treatment could decrease the adipose accumulation in rabbits [7,9]. Acetate attenuates high fat diet-induced body weight/fat gain and insulin resistance in mice [31]. In 3T3-L1 adipocytes, acetate could stimulate adipogenesis [20] and increase the rate of lipolysis [32]. Activation of VFA receptors leads to the inhibition of lipolysis and decrease of plasma free fatty acids [33]. These *vitro* study results agree with our present results that acetate treatment decreased the lipid droplet deposition. The different results of acetate in regulating lipid deposition *in vivo* and *vitro* studies imply that the regulating mechanism is complex. In adipose tissue, Liu et al [7] found that the mediating effect of acetate on lipid deposition is dependent on the dose. A low dose of acetate increased lipid deposition, but a high dose of acetate led to an oppose effect.

The decreased lipid concentration after acetate treatment may be related to increasing HSL. Studies provided evidence that HSL appeared to be the rate-limiting enzyme for cholesteryl ester and diacylglycerol hydrolysis in adipose tissue and is essential for complete hormone stimulated lipolysis [34]. Increased HSL expression after acetate treatment may be an important reason for lower lipid concentration in rabbit ADSCs. Inconsistent with this, a low dose of acetate treatment (6 mM) for a short time (6 h) inhibited HSL expression and increased the lipid deposition in rabbit ADSCs [21]. These results suggest that HSL is a key target of acetate regulating lipid metabolism. In addition, the regulating of acetate in HSL expression and lipid metabolism is associated with the treatment time and dose. In the present study, other tested genes (e.g., *PPARγ*, *C/EBPα*, *FAS*, *ACC1*, and *CPT1*) were not significantly altered after acetate, indicating that these genes may be not the major targets in acetate regulating lipid metabolism in rabbit ADSCs.

### ERK is associated with suppressed lipid deposition by acetate

ERKs are required for the differentiation of 3T3-L1 fibroblasts to adipocytes [35]. The adipocyte-specific transcription factor PPARγ is a substrate of ERK1/2 [36]. In the present study, activation of ERK with ceramide C6 increased *PPARγ* gene expression, suggesting that PPARγ plays a key role in ERK-stimulated adipocyte differentiation. Additionally, activation of ERK increased the *ACC1* and *FAS* genes expression and decreased the *CPT1* gene expression, which suggested that ERK activation could improve the process of fatty acids synthesis and decrease the process of β-oxidation of long-chain fatty acids. Oil red staining results shown ERK activation could increase the lipid deposition, which may be related to the changed process of adipocyte differentiation and fatty acid metabolism.

Acetate decreased the phosphor-ERK1/2 protein level in line with the *vivo* experiment [9], which implies that ERK1/2 signaling was associated with acetate-decreased lipid deposition. The activation of ERK1/2 signaling with ceramide C6 significantly attenuated the acetate-associated decrease in intracellular lipid concentration, demonstrating that ERK1/2 signaling participate in the process of acetate in regulating lipid metabolism. The previous study shows that ERK pathway appears to be able to regulate the process of lipolysis by HSL in adipocytes [37]. Although activation of ERK1/2 with ceramide C6 treatment did not significantly affect the HSL expression, pre-treatment with ceramide C6 significantly attenuated the acetate-stimulated HSL expression. The results suggest that ERK-HSL signaling may be an important regulating pathway of acetate in lipid metabolism. Although *CPT1* gene expression significantly altered after acetate treatment, acetate attenuated the depression of CPT1 caused by ceremide C6, indicating that acetate could recover the decreased process of β-oxidation of long-chain fatty acids caused by activated ERK1/2. Ceremide C6 strongly stimulated C/EBP expression in the presence of acetate, implying that ceremide C6 and acetate could add up to promote the process of adipocyte differentiation. But the mechanism of ceremide C6 and acetate regulating *C/EBP* gene expression is not sure.

Previous studies have shown the p38 MAPK and JNK signaling pathways are involved in the lipid metabolism [18]. But acetate treatment did not significantly affect the phosphorylated protein of p38 MAPK and JNK, indicating that p38 MAPK and JNK signaling pathways maybe not participate in the process of acetate inhibiting lipid accumulation.

### mTOR is associated with suppressed lipid deposition by high dose acetate

As a serine/threonine kinase, mTOR mediates broad biological activities that include translation initiation, transcription, cytoskeleton organization, cell growth, and proliferation as well as cell survival [38]. Recent research showed that mTOR plays an important role in the synthesis and secretion of triacylglycerol [39]. Inhibition of mTOR signaling reduced intracellular lipid accumulation in goose and fish hepatocytes and 3T3-L1 adipocytes [39,40,41]. In our present study, activation of mTOR increased intracellular lipid accumulation in rabbit ADSCs. Activation of mTOR up-regulated the expression of C/EBPα and FAS, and down-regulated the *HSL* gene expression in rabbits ADSCs, suggesting that the activation of mTOR maybe promote the process of adipocyte differentiation and fatty acid synthesis and inhibit the process of lipolysis.

Acetate treatment decreased the phosphor-mTOR protein level, which implies that mTOR signaling maybe take part in the regulation process of acetate in lipid metabolism. To verify our inference, an experiment was conducted involving activated mTOR. Pre-activated mTOR significantly attenuated the stimulation of *HSL* gene expression and the decrease of intracellular lipid accumulation compared to the acetate group. The results demonstrated that acetate could regulate *HSL* gene expression via mTOR. Moreover, mTOR-HSL signaling pathway may be involved in acetate-associated decrease in lipid deposition.

## CONCLUSION

Acetate decreased the lipid deposition and increasing *HSL* gene expression in rabbit ADSCs, and mTOR and ERK1/2 signaling were involved in the process.

## Figures and Tables

**Table 1:** Gene-specific primers used for the analysis of rabbit gene expression

Gene	GenBank accession number	Primers sequences (5′→3′)	Product size (bp)
*GAPDH*	NM_001082253	F: TGCCACCCACTCCTCTACCTTCG R: CCGGTGGTTTGAGGGCTCTTACT	163
*β-actin*	NM_001101683.1	F: CGCAGAAACGAGACGAGATT R: GCAGAACTTTGGGGACTTTG	168
*PPARγ*	NM_001082148.1	F: GGAGCAGAGCAAAGAAGTCG R: CTCACAAAGCCAGGGATGTT	111
*C/EBPα*	XM_008257272.1	F: GTCTACGCTCCACCACCATT R: CCAAACCAGAAGGAAAGAGG	127
*CPT1*	XM_002724092.2	F: ATTCTCACCGCTTTGGGAGG R: ACGGGGTTTTCTAGGAGCAC	196
*FAS*	KF201292.1	F: ACCACGTCCAAGGAGAGCA R: AGTTCTGCACCGAGTTGAGC	112
*ACC1*	XM_002719077.2	F: GTGGTCTTCGTGTGAACTGG R: TTCTTCTGCTGCCTTTAGCC	122
*HSL*	XM_008249691.2	F: CCAGGCTAAACTCGCATCCA R: ATTTGGCTCTCTGGACTGGC	119

*GAPDH*, glyceraldehyde 3-phosphate dehydrogenase; *PPARγ*, peroxisome proliferator-activated receptor-γ; *C/EBPα*, CCAAT/enhancer-binding proteins (C/EBPs); *CPT1*, carnitine palmitoyl transferase 1; *FAS*, fatty acid synthase; *ACC1*, acetyl-CoA carboxylase 1; *HSL*, hormone-sensitive lipase.
