# Supplementary material for: Mechanistic target of rapamycin and an extracellular signaling-regulated kinases 1 and 2 signaling participate in the process of acetate regulating lipid metabolism and hormone-sensitive lipase expression
Source: Anim Biosci. 2021 Oct 29;35(9):1444–53. doi: 10.5713/ab.21.0341 (PMC9449403; doi:10.5713/ab.21.0341)

**Supplementary Figure S1**

The volume density (Vv×200) of Oil Red O-positive rabbit ADSCs after acetate treatment in Experiment 1 (A), ERK1/2 signaling activator (Ceramide C6) and acetate treatments in Experiment 2 (B), or mTOR signaling activator (MHY1485) and acetate treatments in Experiment 3 (C).

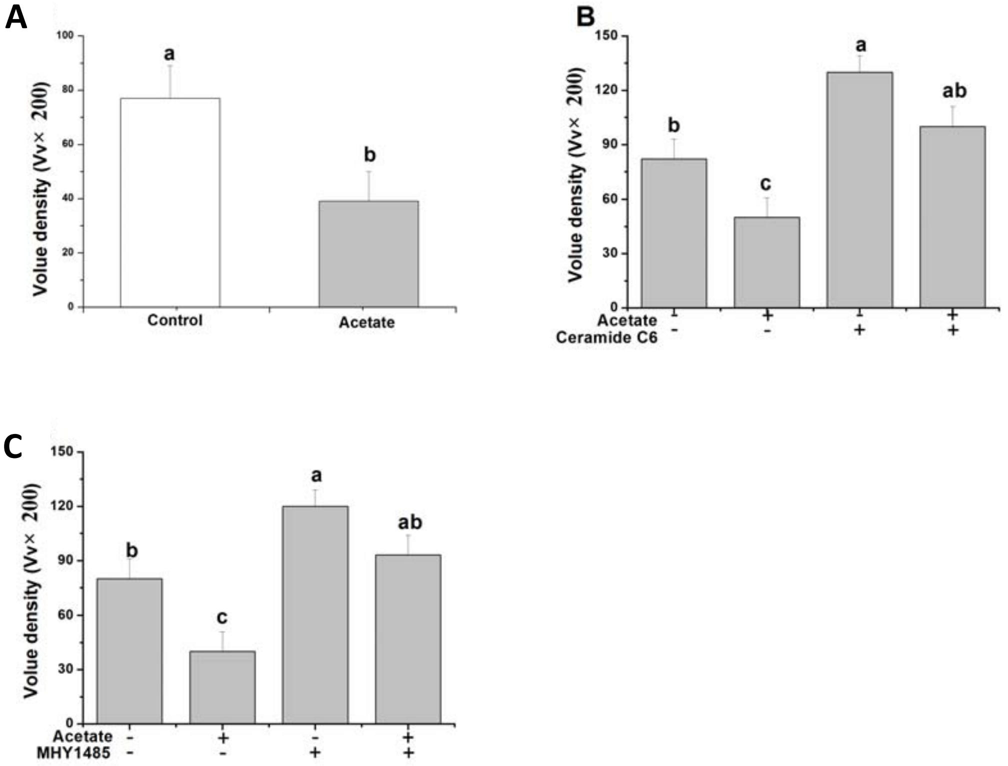

Supplement: Supplementary file 1 [file ab-21-0341-suppl.pdf]
